# Time-Resolved Synchronous Fluorescence for Biomedical Diagnosis

**DOI:** 10.3390/s150921746

**Published:** 2015-08-31

**Authors:** Xiaofeng Zhang, Andrew Fales, Tuan Vo-Dinh

**Affiliations:** 1 Department of Radiology, Duke University Medical Center, Durham, NC 27710, USA; 2 Fitzpatrick Institution for Photonics, Duke University, Durham, NC 27708, USA; 3 Department of Biomedical Engineering, Duke University, Durham, NC 27708, USA; E-Mail: Andrew.Fales@duke.edu; 4 Department of Chemistry, Duke University, Durham, NC 27708, USA

**Keywords:** synchronous fluorescence, ultrafast, time-resolved, imaging, cancer diagnosis

## Abstract

This article presents our most recent advances in synchronous fluorescence (SF) methodology for biomedical diagnostics. The SF method is characterized by simultaneously scanning both the excitation and emission wavelengths while keeping a constant wavelength interval between them. Compared to conventional fluorescence spectroscopy, the SF method simplifies the emission spectrum while enabling greater selectivity, and has been successfully used to detect subtle differences in the fluorescence emission signatures of biochemical species in cells and tissues. The SF method can be used in imaging to analyze dysplastic cells *in vitro* and tissue *in vivo*. Based on the SF method, here we demonstrate the feasibility of a time-resolved synchronous fluorescence (TRSF) method, which incorporates the intrinsic fluorescent decay characteristics of the fluorophores. Our prototype TRSF system has clearly shown its advantage in spectro-temporal separation of the fluorophores that were otherwise difficult to spectrally separate in SF spectroscopy. We envision that our previously-tested SF imaging and the newly-developed TRSF methods will combine their proven diagnostic potentials in cancer diagnosis to further improve the efficacy of SF-based biomedical diagnostics.

## 1. Introduction

### 1.1. Synchronous Fluorescence

Optical technologies for rapid diagnosis of cancer and dysplasia are highly beneficial in early detection and timely treatment. In conventional diagnostics, a biopsy sample typically represents only a very limited area of the suspected tissue; and laboratory results using histopathology examinations are generally time-consuming. Fluorescence spectroscopy is a powerful technique that can be used to noninvasively analyze the fluorescent signatures of tissue. Autofluorescence of neoplastic and normal tissues using fixed-wavelength laser-induced fluorescence (LIF) have been investigated and used for cancer diagnosis in our laboratory as well as other laboratories [1,2,3,4,5,6,7]. Although studies have demonstrated reasonably good specificity and sensitivity in sample classification, fixed-wavelength excitation typically produces fluorescence spectra exhibiting featureless profiles or broad-band peaks, which do not fully exploit the diagnostic potentials of fluorescence spectroscopy. We have previously developed the theoretical foundations of the synchronous fluorescence (SF) method, which was characterized by simultaneously scanning both the excitation and emission wavelengths, while maintaining a constant wavelength interval [8,9]. The SF method has been coupled with multi-component analysis algorithms to obtain spectral signatures of environmental samples and to enhance selectivity in analyzing complex chemical and biological samples [9,10,11,12,13,14].

The SF method not only simplifies the emission spectrum, but is also less affected by Rayleigh and Raman scattering, compared to the conventional excitation-emission matrix fluorescence (EEMF) [15]. The SF methodology provides a simple way to rapidly measure fluorescence signals and spectral signatures of complex biological samples. Conventional fluorescence spectroscopy uses either a fixed-wavelength excitation (*λ*_ex_) to produce an emission spectrum or a fixed-wavelength emission (*λ*_em_) to record an excitation spectrum. With synchronous spectroscopy, the fluorescence signal is recorded while both *λ*_em_ and *λ*_ex_ are scanned simultaneously. A constant wavelength interval Δλ is maintained between the excitation and emission wavelengths throughout the spectrum, as expressed by:

(1) $$\lambda_{em}=\;\lambda_{ex}+\Delta \lambda$$

As a result, the intensity of synchronous signal *I_s_*, can be written as the product of three functions [8,9]:

(2) $$I_{s}\left(\lambda_{ex},\lambda_{em}\right)=k\;c\;E_{x}\left(\lambda_{ex}\right)F_{e}\left(\lambda_{ex},\lambda_{em}\right)E_{m}\left(\lambda_{em}\right)$$

where *k* is a constant dependent on the measurement geometry; *c* is the fluorophore concentration; *E_x_* is the excitation absorption spectrum; *E_m_* is the fluorescence emission spectrum; and *F_e_* is the fluorescence efficiency describing the ratio of excitation light converted to fluorescence. If the wavelength interval Δ*λ* is chosen properly, the resulting SF spectrum will show one or a few features that are much more resolvable than those in the conventional fluorescence emission spectrum.

**Figure 1:**
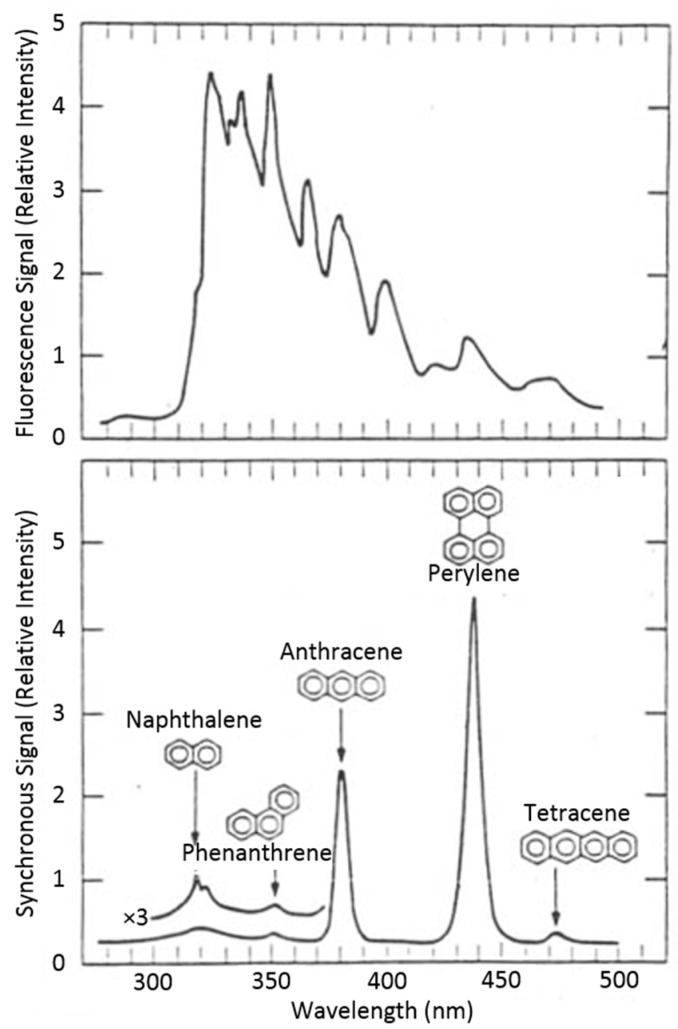
Conventional fixed-excitation fluorescence of a five-component mixture (upper curve, 258 nm excitation) and the SF spectrum of the same mixture showing improved spectral component separation (lower curve, 3 nm wavelength interval). Adapted from [8].

For a single molecular species, the observed SF signal intensity *I_s_* is simplified (often to a single peak) and the bandwidth is narrower than in a conventional fluorescence emission spectrum. This feature significantly reduces spectral overlapping in multicomponent samples. This advantage can be well-demonstrated by the SF spectrum of a sample consisting of structurally-similar compounds: naphthalene, phenanthrene, anthracene, perylene, and tetracene, (Figure 1) [8], in which each compound gives practically only one “peak”, similar to a chromatogram (*i.e.*, a spectrogram).

The SF method has found numerous applications in spectral analysis of complex samples, e.g., in environmental protection [16,17,18], food science [19,20,21], biological assays [22,23,24,25,26,27,28], and medical diagnosis [29,30,31]. In our laboratory, the SF method has been the basis for development of various instruments, including a portable field monitor [10] and an acousto-optic tunable filter (AOTF) system [11]. Most of the early applications of the SF method were focused on examining *in vitro* samples, e.g., analyzing air particulates [32], screening metabolites of a carcinogenic compound, benzo[a]pyrene, characterizing animal and human urine [12], determining carcinogen-macromolecular adducts [33,34], measuring cellular mitochondrial uptake of Rhodamine 123 [35], differentiating normal and neoplastic cells [36], investigating lysozyme-chitobioside interactions [37], studying non-calcium interactions of Fura-2 sensing dye [38], and detecting multiple fluorescent probes in DNA-sequencing [39]. Recently, the SF method has received increased interest in optical diagnosis of cancer. Several other research groups have carried out SF spectroscopy on *ex vivo* tissue samples, such as the cornea [40]. SF has been used to analyze normal and abnormal cervical tissues [41], and three-dimensional, total synchronous luminescence spectroscopy criteria for discrimination between normal and malignant breast tissue [42]. Moreover, the SF method has been used to characterize DMBA-TPA-induced squamous cell carcinoma in mice *in vivo* [43]. Data from total synchronous fluorescence spectroscopy measurements of normal and malignant breast tissue samples were introduced in supervised self-organizing maps, a type of artificial neural network, to obtain diagnosis [30]. Synchronous fluorescence spectroscopy was used for the detection and characterization of cervical cancers *in vitro* [31]. The SF technique has been applied on a variety types of skin tissue to show its narrow-band features and selectivity for *in vivo* analysis [44], and has been applied to both *in vitro* and *in vivo* imaging for cancer detection and diagnostics [30,31,41,45,46,47]. The recent surge of applications has highlighted the unique advantages of the SF method that offers a simple, yet effective, way to capture the fluorescent signatures of complex biochemical compounds in tissue for medical diagnostics.

### 1.2. Synchronous Fluorescence Imaging

**Figure 2:**
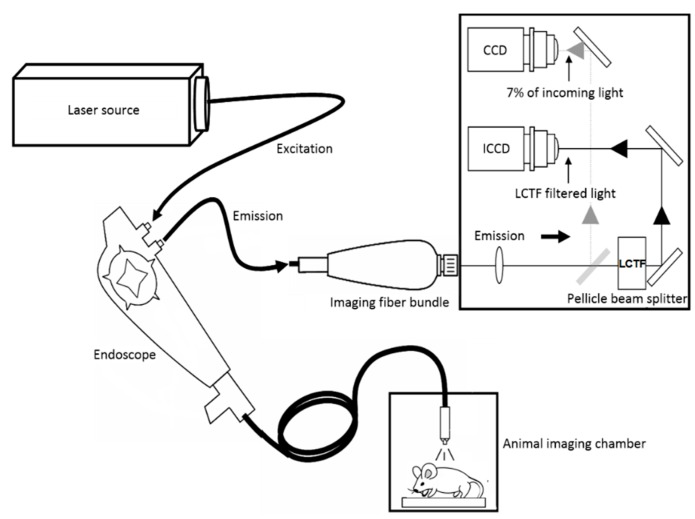
Schematic of the synchronous fluorescence imaging system. Adapted from [45].

We have previously developed a synchronous fluorescence imaging (SFI) system to combine the great diagnostic potentials of the SF method and the large field-of-view of imaging in cancer diagnosis [45,46]. The SFI system can be incorporated into an endoscope for gastrointestinal cancer detection, as schematically shown in Figure 2.

A discriminant analysis and a multivariate statistical method were developed to differentiate pixels containing malignant and normal tissue. MATLAB (MathWorks, Natick, Massachusetts) programs were implemented to allow manual selection of the training pixels for a chosen classifier. The trained classifier would subsequently generate a diagnostic image indicating the condition of each pixel. Both the SF images and the whole set of fluorescence images were analyzed using these two methods. The specificity and sensitivity of the diagnostic image derived from the synchronous data were calculated, using the diagnostic image computed from the full spectral data as the gold standard. Classification results based on the SF data achieved good accuracy compared to the results based on the full spectra when the wavelength interval was chosen appropriately. We emphasize that an attractive advantage of SFI is that a much smaller data set is acquired without losing analytical specificity. Furthermore, the contrast between fluorescence peaks and background in the spectrum is significantly enhanced, which will benefit classification that relies on the subtle spectral differences between malignant and normal tissues. The SFI method, thus, dramatically reduces data acquisition time while maintaining a high classification accuracy, with diagnostic sensitivity from 82% to 97%, depending on the experimental conditions [45,46].

## 2. Methods

### 2.1. Time-Resolved Synchronous Fluorescence

Despite its proven success in comparison to conventional fluorescent methods, the SF method still encounters difficulties in analyzing highly-complex fluorescent samples, where spectral overlapping of multiple fluorophores is severe. This limitation is especially detrimental when both absorption and emission spectra of the fluorophores are closely located. We propose to solve this problem by adding an additional dimension of measurement to the SF method: *i.e.*, using the time-domain information to further separate spectrally-similar fluorophores. The time-domain information of fluorophores is obtained by measuring time-resolved fluorescence decay, which is characteristic to the fluorophores, and, more importantly, is practically uncoupled to the spectral domain information. In our previous work, using an analog time-gating device, we showed improved spectral separation of complex samples [48]. The proposed method significantly improves spectral separation by obtaining the complete time-domain data in just one acquisition cycle.

Time-resolved photo-detection can be achieved using either analog or photon-counting methods. The analog method uses a photo-sensitive device to convert the photon flux to an electric current or voltage, which is subsequently quantized by an analog-digital convertor. Representative analog photodetectors are alkaline-metal photomultiplier tubes and semiconductor photodiodes. The analog method is easy to implement and has been widely used in many applications. However, it has notable limitations in detection sensitivity, response time, output linearity, and device aging. The photon-counting method is established on a completely different principle: it detects the arrival time of individual photons, also known as photon-tagging, and builds the temporal response curve by repeating the photon-tagging operation for a large number of times to create a histogram of the accumulated events of single-photon detection. In this method, the gain of the photosensor is set to a very high level disregarding the linearity range of the device. By doing so, it achieves single-photon sensitivity. The photon-tagging module determines the arrival time of photons with respect to the reference signal fed directly from the pulsed laser source. Compared to the analog method, the photon-counting method dramatically mitigates the limitations on detection sensitivity, response time, output linearity, and device aging.

In the present work, we used the photon-counting method with the single-photon avalanche diodes (SPADs) as the photosensors. With single-photon sensitivity, this configuration was well suited for low-light photodetection. Another important advantage of the photon-counting method is that its temporal resolution is no longer limited by the response time of the photosensor because the temporal response curve is the statistic of the arrival time of the photons, *i.e.*, the electric signal pulses generated by the photosensor in response to the incoming photons. Therefore, the delay due to response time of the photosensor is constant and can be subtracted from the final result collectively with other sources of delay, e.g., optical and electric transmission. Since the significant event is the arrival of photons, it is the timing and number of pulses, rather than the amplitude, that determine the output in photon-counting. Therefore, the linearity range of the photosensor is no longer a concern. In addition, the output linearity is highly stable compared to the analog method and do not require any calibration by the users. Finally, device aging would have virtually no impact on the performance of the photon-counting method because the photosensor produces binary signals whose amplitude is insignificant for the downstream photon-counter. It should be noted that a common limitation of the photon-counting method is the so-called “photon pile-up” effect, which is negligible under the low-light condition.

### 2.2. System Configuration

**Figure 3:**
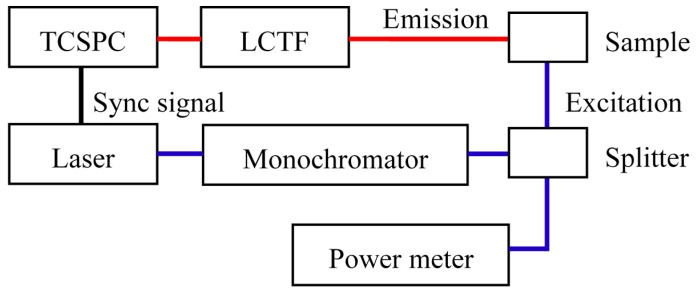
Schematic of the time-resolved synchronous fluorescence (TRSF) system.

Here we report the first prototype system for a TRSF spectroscopy study. The system is schematically shown in Figure 3. A pulsed supercontinuum laser (SC400-2, Fianium, Southampton, UK) was used as a broadband light source: Laser emission between 400 and 2400 nm with an average power density of ~1 mW/nm with a beam diameter of ~1.5 mm in the visible range of 400–700 nm, at a repetition rate of 20 MHz and a typical pulse width of 10 ps. For fluorescence excitation, a short-pass filter (cutoff at 850 nm) was inserted to prevent infrared sample heating. A monochromator (DMC1-03, Optometrics, Littleton, MA, USA) was used to adjust the excitation wavelength. On the emission side, a liquid-crystal tunable filter (LCTF, VariSpec VIS-20, PerkinElmer, Waltham, MA, USA) was used as a band-pass filter tunable between 400 and 720 nm. Time-resolved photodetection was achieved using a time-correlated single-photon counting (TCSPC) module (SPC-150, Becker and Hickl, Berlin, Germany) coupled with an SPAD (Micro Photon Devices, Bolzano, Italy).

It is noteworthy that the proposed TRSF methodology can be easily translated to imaging applications, for example, by coupling the photodetector to a wide-field imaging lens and performing raster scans in the imaging plane of the lens.

## 3. Results and Discussion

**Figure 4:**
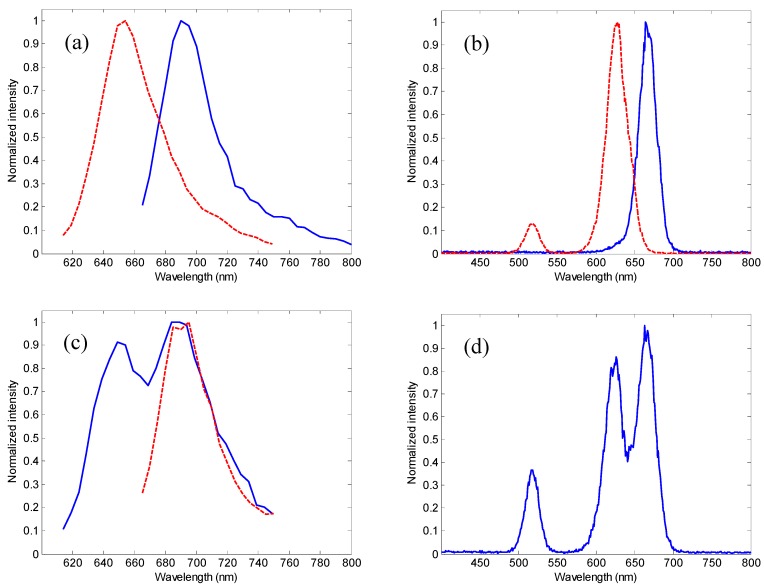
Fluorescence and standard SF spectra of methylene blue, oxazine 170, and their mixture. (**a**) conventional fluorescence emission spectra of methylene blue (blue solid line) and oxazine 170 (red broken line); (**b**) standard SF spectra of methylene blue (blue solid line) and oxazine 170 (red broken line); (**c**) conventional fluorescence spectra of the mixture of methylene blue and oxazine 170, excited at 614 nm (blue solid line) and 655 nm (red broken line); and (**d**) standard SF spectrum of the mixture of methylene blue and oxazine 170 (wavelength interval of 20 nm).

To demonstrate the proposed TRSF system, we used a sample consisting of methylene blue and oxazine 170 (both 10 µM) in deionized water. Methylene blue is frequently used as a dye to stain certain types of tissue during surgery, and can be used as an injectable to treat methemoglobinemia [49]. Oxazine 170 is an important laser dye and has recently been demonstrated in a feasibility study as a nerve-specific marker for neurosurgery [50]. These two fluorophores have substantially overlapping absorption and emission spectra that render SF spectral separation ineffective, shown in Figure 4, which were acquired using a commercially available spectrometer (Jobin Yvon FluoroMax 3, Horiba Scientific, Edison, NJ) with both excitation and emission slit widths set to 1 nm. The spectral sweeping step size was 5 nm for fluorescence (Figure 4a,c) and 1 nm for SF (Figure 4b,d). The wavelength interval of the SF scans was 20 nm.

It is apparent in Figure 4c,d that spectral separation of methylene blue and oxazine 170 in the SF spectrum is significantly better than in conventional fluorescence spectrum. Nonetheless, we emphasize that the SF spectrum was acquired with a slit width of 1 nm and a sweeping step of 1 nm, which may be impractical in many applications, e.g., multispectral imaging or fast acquisition, when the spectral pass-band widths and sweeping step are significantly broader (typically 10 nm or higher). Under these circumstances, spectral separation may become unsatisfactory, which is shown below.

Using our newly-developed prototype system, we obtained both SF and TRSF spectra of the same set of samples consisting of methylene blue and oxazine 170 (freshly prepared). The excitation and emission wavelengths were adjusted by tuning the monochromator and the LCTF simultaneously between consecutive data acquisitions with a wavelength interval of 40 nm. We chose this wavelength interval because the optical density of this particular LCTF is the smallest at 40 nm within the range of typical interval values (20–60 nm). Note that the bandwidths of the LCTF and monochromator were 20 and 10 nm, respectively, limiting the smallest allowable wavelength interval for this configuration to 15 nm. Considering the transition bandwidth of the devices, the minimal practical value of the wavelength interval would be ~20 nm.

**Figure 5:**
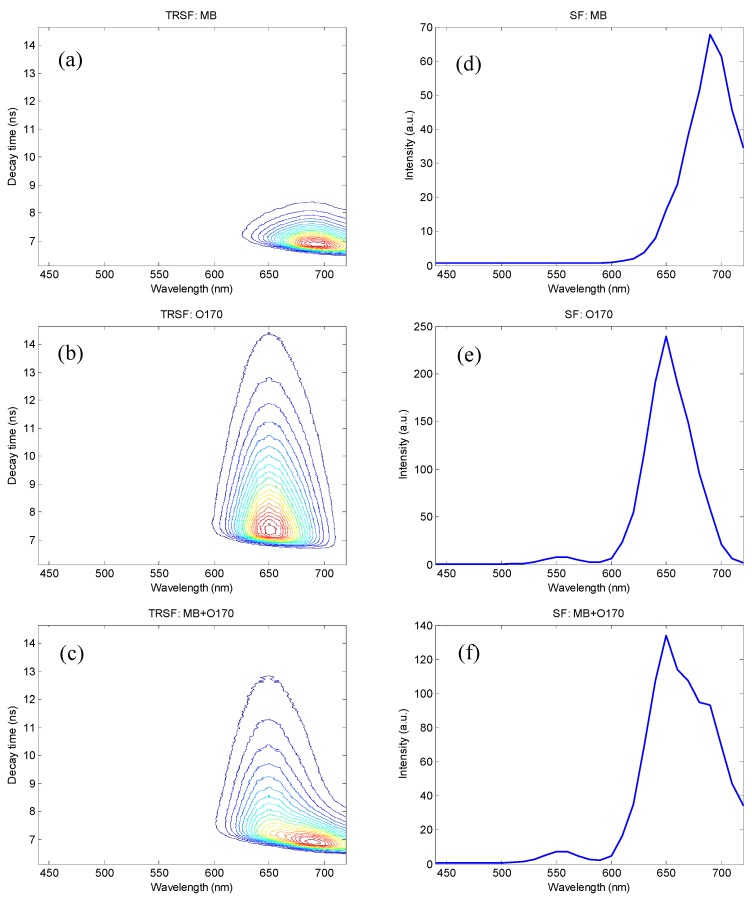
SF and TRSF spectra acquired using the TRSF system: linear contour plots of the TRSF spectra of (**a**) methylene blue (MB); (**b**) oxazine 170 (O170); (**c**) their mixture (MB + O170); and (**d**–**f**) SF spectra derived from the same data sets, respectively.

The TRSF and SF spectra acquired by the new system are shown in Figure 5. The TRSF spectra (Figure 5a–c) were acquired by plotting the photon count with respect to the photodetection/emission wavelength (the x-axis) and the photon time-tag (the y-axis). The SF spectra (Figure 5d–f) were obtained by disregarding the photon time-tag (*i.e.*, collapsing the y-axis). The spectral range (excitation) was 400–680 nm, which was jointly defined by the laser spectrum (400–850 nm) and the tuning range of LCTF (400–720 nm). The corresponding spectral range of the TRSF and SF scans was 440–720 nm.

**Figure 6:**
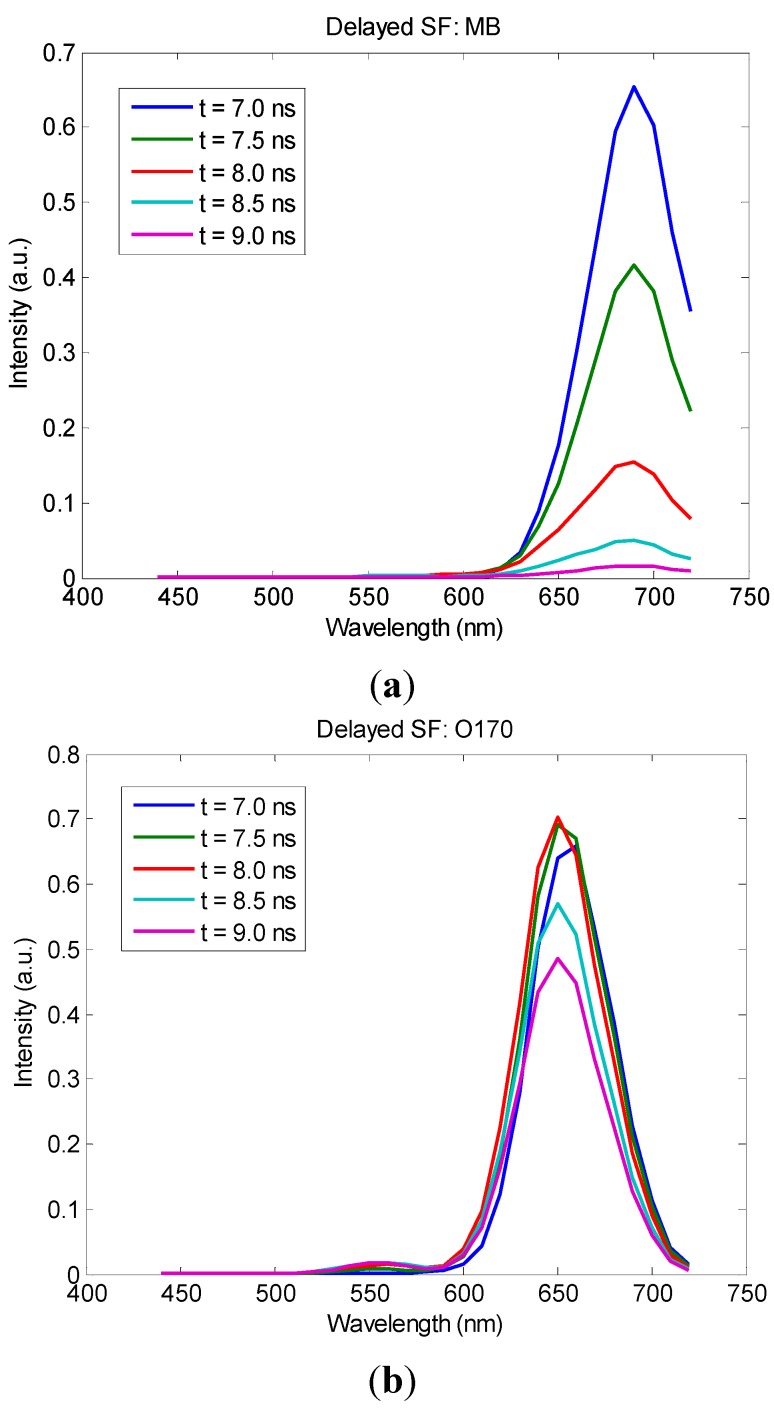

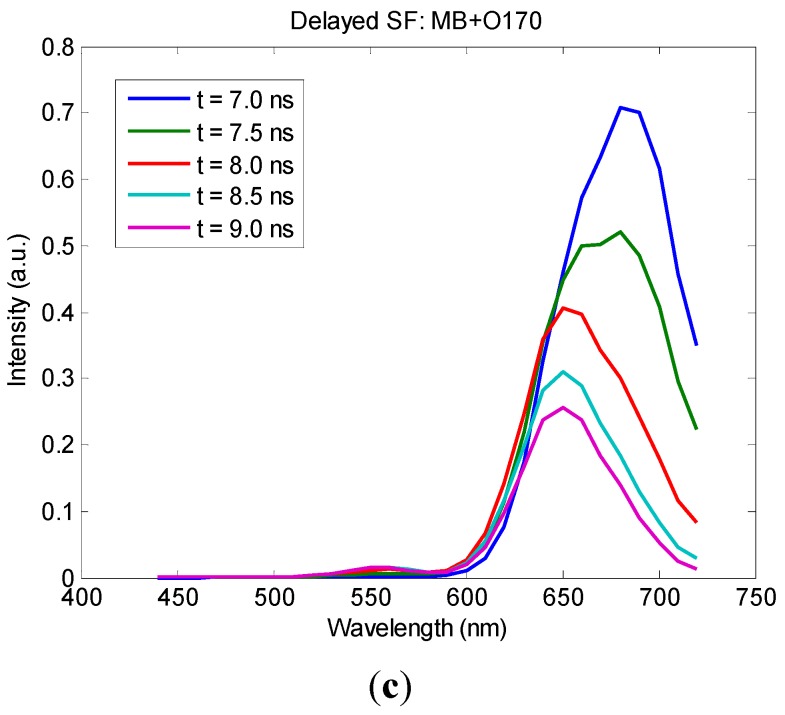
Time-delayed SF (“Delayed SF”) plots derived from the TRSF data set of (**a**) methylene blue (MB); (**b**) oxazine 170 (O170); and (**c**) the mixture of the two fluorophores (MB + O170).

It was apparent that, using the SF method, methylene blue and oxazine 170 could not be spectrally resolved with the given excitation/emission configuration, Figure 5f. This was mainly because of peak-broadening due to differences in system configuration and device specifications, comparing Figure 4d and Figure 5f. The difference in fluorescence quantum efficiency is another complicating factor, referring to the spectra obtained by the same instrument shown in Figure 5d–f. Both of these factors have made effective spectral separation of the two fluorophores impractical. With the time-resolved data shown in Figure 5c, however, clear distinction between the two fluorescent species is evident, comparing Figure 5c to Figure 5a,b. Specifically, single-component samples exhibit highly symmetric patterns (Figure 5a,b) because of the symmetric fluorescent emission peaks of the individual fluorophores. In contrast, the two-component sample produced a highly asymmetric pattern (Figure 5c) because fluorescence lifetime constants of the constituent fluorophores are different. In other words, one observes a shifting fluorescent emission peak at different time points of fluorescence decay, which can be better illustrated by plotting the SF spectra of the set of samples at different time points, Figure 6.

The existence of shifting peak wavelength in the “delayed SF” spectra can be used as a quantitative indicator in automatic multi-component fluorophore identification. With the additional time-resolved information, the lifetime constants of the fluorophores can be used in spectro-temporal analysis. Similar to the extension of the SF method to SFI, the concept of TRSF can also potentially be extended to biomedical imaging. Note that the TCSPC acquisition method is based on photon-counting and that it does not perform additional scans (e.g., time-binning) in the time-domain. For this reason, TRSF imaging will not increase acquisition time except for the intrinsic signal-to-noise limit. Furthermore, in analyzing complex biomedical specimens, high-dimensional analysis that involves spectral, temporal, and spatial information will likely give more robust classification results than low-dimensional analysis does. We are currently working toward TRSF imaging instrumentation and multi-dimensional data analysis algorithms for biomedical applications.

## 4. Conclusions

Synchronous fluorescence (SF) is a fast and information-rich spectroscopy method. It has unique advantages compared to the conventional fixed-excitation fluorescence spectroscopy. Based on the SF method, we demonstrated the feasibility of a new time-resolved SF (TRSF) method and instrument. This method is a significant advancement from our previous work that revealed differences in spectral data with different acquisition time-delay. In the present work, a complete dimension in the time-domain is added to the spectral data, which is conveniently obtained in one acquisition cycle. Compared to the standard SF method and the previous method, the proposed TRSF method better captures the full characteristics of fluorophores in complex samples. Using a two-fluorophore liquid sample as an example, our prototype TRSF system clearly showed its advantage in spectro-temporal separation of the fluorophores that were otherwise difficult to resolve with conventional SF spectroscopy. Although application of the proposed method on actual biological samples is beyond the scope of the present work, we envision that our previously-tested SFI and the newly-developed TRSF methods can combine the impressive diagnostic potentials of the SF spectroscopy, wide-field imaging, and the new ultrafast time-resolved acquisition, and that the new technologies will further improve the selectivity of fluorescence-based analysis and diagnostics. Lastly, we would like to emphasize that SF is a methodology for rapid data acquisition, which can be potentially applied to a number of analytical and imaging technologies to achieve superior performance that were previously unattainable.
